# Multifaceted Nature of DNA Polymerase θ

**DOI:** 10.3390/ijms24043619

**Published:** 2023-02-10

**Authors:** Alexander A. Kruchinin, Alena V. Makarova

**Affiliations:** National Research Center “Kurchatov Institute”, Institute of Molecular Genetics, Kurchatov Sq. 2, 123182 Moscow, Russia

**Keywords:** DNA polymerase θ, double-strand break repair, DNA translesion synthesis

## Abstract

DNA polymerase θ belongs to the A family of DNA polymerases and plays a key role in DNA repair and damage tolerance, including double-strand break repair and DNA translesion synthesis. Pol θ is often overexpressed in cancer cells and promotes their resistance to chemotherapeutic agents. In this review, we discuss unique biochemical properties and structural features of Pol θ, its multiple roles in protection of genome stability and the potential of Pol θ as a target for cancer treatment.

## 1. Introduction

Genome stability is one of the highest priorities of any organism. It can be achieved in different ways, combined in one general metabolic pathway known as the DNA damage response (DDR). It includes mechanisms that carry out both DNA repair and DNA damage tolerance (DDT).

DNA polymerases are enzymes that primarily synthesize complementary DNA strands during replication. Six families of DNA polymerases have been currently identified: A, B, C, D, X and Y. Besides classic DNA polymerases, a DNA primase-polymerase PrimPol, which belongs to the superfamily of archaeo-eukaryotic primases, was described in 2013. The functions of DNA polymerases, however, are not limited to high-fidelity replication (eukaryotic DNA polymerases α, δ, ε from the B family); they also play an important role in DDR pathways, including base excision repair (eukaryotic DNA polymerases β, λ from the X family), double-strand break (DSB) repair (eukaryotic DNA polymerases λ, μ from the X family) and DNA translesion synthesis (TLS) (eukaryotic DNA polymerases of the Y family and DNA polymerase ζ from the B family) [1]. Nevertheless, in cancer cells, these mechanisms often support tumor progression, making them potential targets for therapy.

Eukaryotic DNA polymerases that belong to the A family differ greatly in their functions. Perhaps the most well-known member of this group is DNA polymerase γ, the main player in mitochondrial replication. The functions of the other members, DNA polymerase θ (Pol θ) and DNA polymerase ν (Pol ν), are quite distinct, even though the Pol θ and Pol ν catalytic cores are partially homologous to Pol γ and *Escherichia coli* Pol I.

In 1996, the analysis of *Drosophila melanogaster* mutant alleles of the *Mus308* gene revealed hypersensitivity to cross-linking agents such as photoactivated psoralen, diepoxybutane, and nitrogen mustard. Thus, it was suggested that the *Mus308* gene product should be involved in DNA repair [2]. A central role of Pol θ in DSBs repair, the Pol θ-mediated end joining (TMEJ), was demonstrated later, in 2004, using a mouse model [3]. During TMEJ, Pol θ aligns resected 3′-single-stranded DNA ends based on microhomology, fills DNA gaps and generates repair products with deletions of nonhomologous sequences flanking the DSB site.

As it turned out, Pol θ cell functions are more diverse than previously expected. This unique enzyme is involved in a number of different DNA-related pathways reviewed in [4,5,6,7,8]. In this review, we discuss unique properties of Pol θ, its roles in the protection of genome stability and integrity as well as recent progress in studies of Pol θ as a target for cancer treatment.

## 2. Structure and Domain Organization of Pol θ

**Figure 1:**
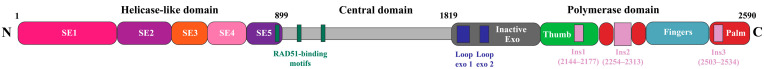
Domain organization of Pol θ. The N-terminal helicase-like domain (HD), the C-terminal polymerase domain (PD), the unstructured central domain (CD), and the exonuclease-like subdomain are shown. The positions of the RAD51-binding motifs, the thumb, fingers and palm subdomains and the Inserts 1–3 are indicated. Structural elements (SE) of the HD are RecA-like subdomain 1 (SE1), RecA-like subdomain 2 (SE2), a winged helix subdomain (SE3), a helical bundle (SE4), and a helix-hairpin-helix subdomain (SE5). The structural element borders are indicated according to [10,11].

*POLQ*-like genes are widely represented among multicellular eukaryotic organisms, including plants and protists. However, among fungi, including yeast, *POLQ*-like genes are not identified [9]. Human Pol θ is a 290 kDa protein which consists of 2590 amino acid residues arranged in three domains (Figure 1).

Unlike other DNA polymerases, Pol θ contains a separate superfamily 2 (SF2) helicase-like domain (HD) located on the N-terminus of the protein [12]. Crystal structures of the separate SF2 HD and polymerase domain (PD) of human Pol θ have been solved [10,11]. The high-resolution cryo-EM structure of the PD of *Lates calcarifer* Pol θ is also available [13]. The HD is composed of five structural elements. Among them are the nucleotide-binding site and two RecA-like subdomains (residues 1–289 and 290–513) containing motifs I–VI, which are conserved across SF2 helicases. The HD sequence similarity is observed among *Homo sapiens*, *Mus musculus*, *D. melanogaster*, *Caenorhabditis elegans*, and even archaea [10]. DNA-dependent ATPase activity in vitro is associated with the HD [12]. Purified HD is able to unwind different types of DNA with 3′-5′ polarity (in an ATP-dependent manner) and reanneal them [14,15]. Importantly, the HD forms a tetramer (a dimer of dimers) both in the crystals and in solution [10], providing the structural basis for the bridging and annealing of DNA strands by Pol θ.

The PD of Pol θ is located on the C-terminus and consists of four subdomains: thumb, palm, fingers and the catalytically inactive exonuclease-like subdomain [11]. Like other eukaryotic members of the A family, the sequence of the PD catalytic core is similar to that of *E. coli* Pol I [2]. The palm subdomain harbors highly conserved (from worms to human) catalytic D2330, D2540 and E2541 residues that coordinate bivalent metal ions [11]. The G2384 and Y2387 residues of the fingers subdomain are responsible for stabilizing the incoming nucleotide. The fingers subdomain is highly flexible and may contribute to Pol θ tolerance to mismatches and bulky DNA lesions [13].

Three conserved loop elements are located within the PD. These insertion loops are not found in the two other A-family polymerases. Insert 1 consists of 31 amino acid residues between the first and second conserved motifs and is located in the thumb subdomain. Its proximity to DNA suggests a role in DNA binding. Insert 2 and Insert 3 are located in the palm subdomain and are comprised of 58 and 33 amino acids, respectively [11]. These elements play a crucial role in the TLS activity and the ability of Pol θ to use primers with only minimal homology to DNA templates during TMEJ [16]. The thumb residues K2181 and R2202 as well as the Insert 2 R2254 residue make unique upstream contacts with the 3′-terminal primer phosphates (n-1–n-5). These contacts can facilitate DNA lesions bypass and the extension of poorly annealed DNA termini during TMEJ. The inactive exonuclease-like subdomain, lacking catalytic residues essential for metal-ion binding and primer degradation, has two conserved loops [11].

The dimerization or oligomerization of the PD was demonstrated in [11,17,18] but was not observed in the cryo-EM structure of the PD of *L. calcarifer* Pol θ [13].

The N-terminal HD and the C-terminal PD are connected by a long unstructured region called the central domain (CD). This region regulates multimerization of Pol θ during TMEJ and is involved in the regulation of substrate selection by Pol θ, preventing TMEJ on short (≤26 bp) single-stranded DNA (ssDNA) substrates [18].

At least three RAD51-binding motifs were found in Pol θ. One of them is located in the HD and the other two in the CD [19]. It was shown that interaction between RAD51 and Pol θ is modulated through the RAD51-binding site in the HD, which is most evolutionarily conserved among others in Pol θ [14].

## 3. Pol θ as a Central Player in TMEJ

### 3.1. Discovery of TMEJ

DSBs arising under various conditions (oxidative and genotoxic stress, ionizing and UV radiation) and accompanied by genomic instability are extremely dangerous to cells. DSBs can lead to malignant cell transformation, cell death, and the development of various pathologies. To withstand this type of damage, the cell relies on at least four major types of DSB repair. The non-homologous end joining (which is now often referred to as canonical NHEJ, or c-NHEJ) does not require a homologous chromosome and directly ligates the break ends. NHEJ is typically guided by very short (1–4 bp) terminal microhomologies but can be realized even without microhomology [20].

Other types of DSB repair pathways—single-strand annealing (SSA), homologous recombination (HR) and alternative end joining (a-EJ)—are based on different principles, which require 5′ to 3′ nucleolytic resection of broken ends to generate 3′-ssDNA tails at the DSB site and more extensive homology to anneal complementary sequences.

At present, the importance of Pol θ for DSB repair is well-established. It took more than a decade to uncover the molecular basis underlying the role of Pol θ in DSB repair. The phenotype-based screening of mutant mice showed that the missense mutant allele *chaos1* (a C>T substitution at nucleotide 5794 in the coding region of exon 19) of the *Polq* gene leads to a spontaneous and radioactivity-induced increase in micronuclei number in erythrocytes. The analysis of *Polq^chaos^*^1^/*Polq^chaos^*^1^ mice with an ATM (ataxia telangiectasia mutated) deficiency allowed the suggestion that Pol θ might be involved in an alternative DSB repair pathway distinctive from the major HR ATM-dependent pathway [3]. About 90% of *chaos*1 homozygous mice with an ATM-deficient background died during the neonatal period and surviving animals exhibited severe growth retardation and chromosome instability which was synergistic to the ATM-deficiency phenotype. A study by J. Goff et al., demonstrated the sensitivity of *POLQ*-defective bone-marrow cells to γ-radiation and bleomycin, suggesting the role of Pol θ in DSB repair [21]. Finally, experiments by S.H. Chan et al., with the P-element of *D. melanogaster* revealed that Pol θ is a critical factor of Lig4 (DNA ligase 4)-independent alternative end joining (a-EJ) [22].

As discussed in [7] by Ramsden et al., previously, a-EJ was considered as a DSB repair pathway which does not depend on LIG4. Currently, the term “a-EJ” is applied to a group of end-joining pathways that do not require at least one of the c-NHEJ factors: Ku, XRCC4 or LIG4. a-EJ is often associated with microhomology-mediated end joining (MMEJ) and DNA polymerase theta (Pol θ)-mediated end joining (TMEJ). Definitions of a-EJ and TMEJ are not equivalent. In fungi, end joining proceeds in a Pol θ-independent manner. Thus, a-EJ can be divided into two subgroups: TMEJ and Pol θ-independent a-EJ (the definition is given based on a key factor involved). In turn, MMEJ is an umbrella term for end joining realized by the mechanism of broken DNA strand alignment based on microhomologous sequences [7].

### 3.2. Mechanism and Regulation of TMEJ in Cells

**Figure 2:**
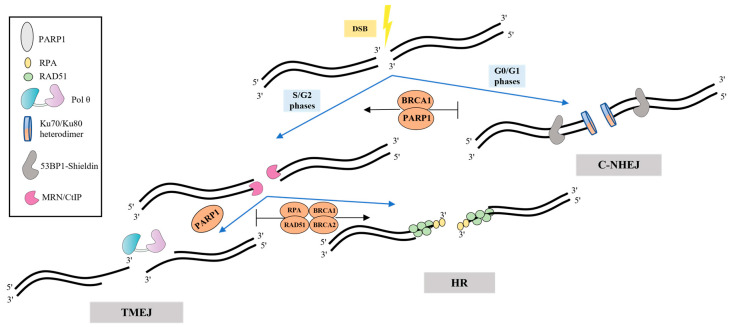
Regulation of TMEJ. Loading of Ku heterodimer, which prevents resection and promotes c-NHEJ, is very effective at blunt DNA ends and extremely short overhangs. Another factor, 53BP1 recruits the shieldin complex inhibiting 5′-3′ resection of DNA ends, thereby promoting c-NHEJ and antagonizing HR and TMEJ. The MRN/CtIP complex performs end resection accompanied by formation of intermediates that are common for both HR and TMEJ. HR factors (RPA, RAD51, BRCA1 and BRCA2) contribute to more accurate repair, inhibiting error-prone TMEJ. PARP1 appears to mediate the recruitment of Pol θ.

Several factors favoring the cell choice of TMEJ have been defined. The main regulation is most likely realized during the first step—resection, when the 3′-ssDNA overhangs are generated by the MRE11–RAD50–NBS1 (MRN) complex and CtBP-interacting protein (CtIP) [7]. The formation of such intermediates, which are unsuitable substrates for the c-NHEJ Ku factors, antagonizes this pathway and favors HR and TMEJ. The phase of the cell cycle, the presence of some regulatory and cell-signaling proteins, and the length and degree of homology of the processed 3′-overhangs appear to be the main factors in the Pol θ recruitment and cell’s choice of TMEJ over c-NHEJ, HR and SSA (Figure 2) [23,24,25,26,27,28,29]. TMEJ, together with HR and SSA, are limited to the S and G2 stages, while c-NHEJ operates predominantly in the G0/G1 phases [29].

TMEJ is also regulated by poly(ADP) ribose polymerase 1 (PARP1). PARP1 is activated upon binding DNA strand breaks and performs post-translational modification (PARylation) of many DNA repair proteins. Competing with Ku proteins for DNA ends binding, PARP1 promotes a-EJ, and its inhibition impairs Pol θ recruitment to DNA break sites [30,31]. Chromosomal TMEJ assays revealed that PARP1/PARP2 deficiency reduces TMEJ activity only 2–4-fold through a decrease in the 5′-3′ resection of DSB, and, thus, re-channels repair to NHEJ [32]. However, PARP1-dependent PARylation of the Pol θ HD in vitro decreases the ssDNA-binding affinity of Pol θ. It was suggested that PARylation can promote the dissociation of Pol θ from DNA strands and termination of DNA synthesis in the end of TMEJ [33]. The alternative clamp RAD9-RAD1-HUS1 complex (9-1-1), Fanconi anaemia group D2 protein (FANCD2) and 5-hydroxymethylcytosine-binding protein (NMCES) possibly also play a role in the recruitment of Pol θ to DNA [reviewed in [7]].

Before binding to DNA, it is necessary to displace replication protein A (RPA), which coats ssDNA regions limiting TMEJ and contributing to HR. Pol θ promotes the removal of RPA from single-stranded overhangs via ATP hydrolysis [14]. Moreover, the assembly of RAD51 filaments on ssDNA sites in order to promote HR can be suppressed by Pol θ. Thus, *POLQ*-knockdown in HR-proficient cells contributes to the RAD51 filaments assembly and accumulation [19,31] Disassembly of RAD51 nucleofilament by Pol θ is carried out in ATPase-dependent manner but with lower efficiency compared to RPA [33].

**Figure 3:**
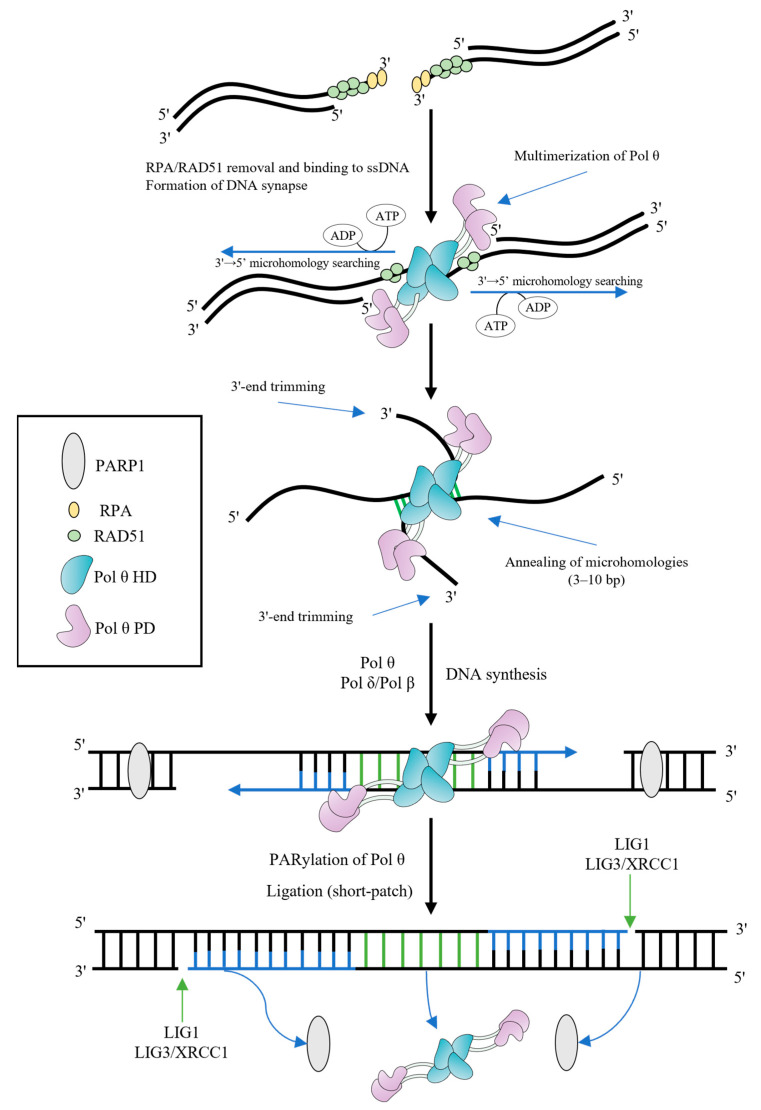
Model of TMEJ. The resected 3′-overhangs are coated by RPA and RAD51 filaments. The HD of Pol θ removes both of them in ATP-dependent manner, contributing to TMEJ. Multimerization of Pol θ promotes the DNA synapse formation and initiates the 3′→5′ bidirectional searching for microhomologies, using the energy of ATP hydrolysis. Aligned 3′-ends are annealed in microhomology-rich regions. Unannealed 3′-overhangs are trimmed before DNA extension. Pol θ (or Pol β) fills gaps from annealed microhomologies, using them as primers. PARP1 PARylates Pol θ in order to remove the polymerase from DNA and complete the synthesis step. LIG1 or LIG3/XRCC1 complex finish the end joining (the short-patch resolution is shown).

The 3′-overhangs lacking RPA should be paired and aligned, followed by the search for microhomologies and annealing (Figure 3). There are a number of different conditions for completing these steps [17,26,28,34]. In vitro assays indicated that the 3′-ssDNA overhangs shorter than 15 nt are favorable for TMEJ [17]. However, the presence of 3′-tails of >45 nt is an important requirement for promoting TMEJ in cells [28].

Some structural studies demonstrated that the HD and PD form multimers regulated by the CD and Insert 2 [10,11,17,18]. Multimerization of Pol θ might contribute to both bridging non-complementary DNA ends to form a DNA synapse, and searching for microhomologies [10,33]. Identification of the site of microhomology relies on a mechanism associated with the HD and involves initiation of a bidirectional search from the 3′-end [34]. As a rule, Pol θ successfully recognizes the microhomology when it is within the 15-nt region on either side of the break site [34], though some works indicated that microhomologies may be at a farther distance from the 3′-overhangs. Microhomologies need to comprise 3–10 bp for effective DSB repair by TMEJ [34].

Aligned 3′-ends are expected to promote the annealing accompanied by predominantly intramolecular hairpin formation rather than intermolecular annealing and DNA synthesis. Nevertheless, the unknown endonuclease activity can cleave hairpin structures that form during intramolecular annealing, facilitating intermolecular annealing as well as multimerization of Pol θ. The Pol θ HD suppresses snap-back replication, favoring intermolecular annealing and synthesis [10,18,35].

The presence of nonhomologous segments flanking the microhomologies at the 3′-ends complicates TMEJ. Nonhomologous regions should be removed and the 3′-tails should be shortened before initiation of DNA synthesis from annealed microhomologies. Initially, it was suggested that Pol θ performs end trimming using the intrinsic endonuclease activity associated with the PD (repurposing of metal ions in the polymerase active site for endonucleolytic cleavage) [35]. However, in a further paper, an alternative explanation of the results was provided which made the authors doubt the existence of trimming endonuclease activity [5]. The PD structure lacks a potential endonuclease domain [11]. Thus, the mechanism of the 3′-end processing is yet to be established. The 3′-flap endonuclease complex excision repair cross complementation group 1-xeroderma pigmentosum group F (ERCC1-XPF) is a possible candidate since the XPF deficiency is associated with a reduced DSB repair efficiency (independently from the NHEJ repair pathway) [36].

After the 3′-end-trimming step, Pol θ initiates template-dependent fill-in DNA synthesis [35]. Annealed microhomologies represent themselves as DNA primers and usually contain fewer than 10 nucleotides. The solved crystal structure of the PD reveals how Pol θ grasps the primer by making contacts with its phosphate backbone [11]. The processive DNA synthesis is important for efficient TMEJ, since the length of the 3′-overhangs before trimming often varies. In fact, the processivity of Pol θ is generally higher as compared to Y-family DNA polymerases. Pol θ is able to incorporate up to 100 nucleotides in one round of template binding in vitro [37,38,39]. Yet, sometimes processivity may not be sufficient during TMEJ (e.g., in the case of an only 1–2 nt microhomology), and it was suggested that Pol θ can carry out DNA synthesis in several rounds of DNA binding [22,28,34,40]. It was shown that the thumb subdomain Insert 1 residues contacting the DNA minor grove are important for processive DNA synthesis by Pol θ [11,16].

The DNA synthesis step is apparently not restricted to Pol θ; the high-fidelity replicative DNA polymerase Pol δ may be involved both in yeast and mammals [41,42]. It has been also hypothesized that, due to insufficient processivity, Pol θ only initiates synthesis from annealed microhomologies, and, subsequently, Pol δ fills the gap. Pol β, which can operate on the 3′-overhangs during gap-filling synthesis in vitro, is considered to be a back-up DNA polymerase for Pol θ [43].

After filling the gap, DNA ligase 1 (LIG1) or (preferentially) the complex of DNA ligase 3 (LIG3)/X-ray repair cross-complementing protein 1 (XRCC1) seals the DNA ends during a short-patch resolution, restoring a phosphodiester bond [22,31]. Alternatively, a DNA polymerase can displace DNA strand and generate a 5′-flap, which is removed by flap endonuclease 1 (FEN1) or DNA2 followed by ligation (a long-patch resolution) [44,45].

## 4. Pol θ Provides Genome (In)Stability

### 4.1. Templated Insertions (TINs) and Deletions—A Unique “Footprint” of Pol θ

TMEJ is associated with specific mutations at the break sites—short insertions (3–10 nt) called templated insertions (TINs) which are unique hallmarks of DSB repair by Pol θ [22,28,34,40,46]. Insertions are identical to the flanking DNA sequences that serve as templates for extensions from microhomologies. The activity of Pol θ correlates with TINs frequency in cancer cells deficient in the HR pathway [34]. Generation of TINs is well-known as a concomitant mutagenic event during the repair of CRISPR/Cas9-induced chromosome breaks via TMEJ [47].

A presumed mechanism of direct TINs generation is interrupted DNA synthesis initiated from aligned microhomologies. Regions with a small degree of microhomologies (<3 nt) and AT-rich regions are associated with unstable alignment and cause abortive enzyme binding to the primer-template during DNA synthesis. Repetitive cycles of re-alignment and DNA synthesis generate direct TINs [34]. Another possible mechanism includes the formation of a loop generated during annealing between misaligned direct repeats [48]. Intramolecular synthesis, which is accompanied by the production of transient secondary hairpin structures and leads to snap-back replication, can result in inverted repeat TINs [34,48]. It is worth noting that TINs formation can serve as an adaptive mechanism. The generation of new microhomologies in microhomology-poor regions promotes successful DNA repair by Pol θ during the next DNA synthesis round (generating a new, larger and more stable microhomology) [34].

Deletions of >1 bp and <30 bp are other types of mutations common for TMEJ. Deletions are associated with microhomologies located within 15 bp flanking the break site (microhomology-associated deletions, MHDs) [34,49]. MHDs are generated during a single round of alignment and processive DNA synthesis from microhomologies followed by the degradation of nonhomologous sequences. Some deletions contain (at the deletion junction) small templated inserts (delins) [34].

### 4.2. Other Pol θ-Associated Mutations

Initially, it was suggested that Pol θ is a relatively accurate DNA polymerase and its error frequency on undamaged DNA was found to be comparable to Pol β and even the high-fidelity Pol α [50]. However, later studies did not confirm the high accuracy of the enzyme. Pol θ does not have the proofreading 3′→5′ exonuclease activity and demonstrates low fidelity despite the high sequence similarity of the active site of Pol θ with other members of the A family [12,37]. The substitution error rate of Pol θ in vitro is 2.4 × 10^−3^, which is comparable to DNA polymerases of the Y family [37]. Pol θ predominantly generates substitutions opposite T, inserting either dTMP or dGMP during primer extension reactions. The primer-template slippage mechanism does not influence the misincorporation rates on undamaged DNA templates [51]. Pol θ also generates indels at homopolymeric runs during gap filling synthesis in a sequence-context-dependent manner [37].

Frameshift mutations arising during TLS can be explained by the unique Pol θ active-site architecture. Insert 2 conserved between Pol θ and Pol ν forms a cavity in the DNA-binding surface, which can accommodate different types of lesions. The lesion becomes extrahelical, and the misalignment is stabilized by the pairing of the incoming dNTP with the complementary next template base, which results in a single-base deletion [52].

Moreover, in human cells, random integration of exogenous DNA during TMEJ and c-NHEJ DSB repair was demonstrated. The off-target integration events are dramatically decreased in *POLQ*-deficient cells, while the gene targeting efficiency, including genome editing by CRISPR/Cas9, are significantly increased [53,54]. Findings of dramatic Pol θ-dependent genome alterations, such as chromosomal translocations, are contradictory [28,31].

### 4.3. Role of TMEJ in Genome Stability

Why does the cell use mutagenic TMEJ? The whole genome sequencing of *C. elegans* propagated for many generations suggests an important role of TMEJ in DSB repair and the prevention of global chromosomal rearrangements [55,56]. *C. elegans* strains defective for Pol θ lack small deletions but spontaneously accumulate more dramatic chromosomal rearrangements.

Pol θ deficiency has no immediate dramatic consequences itself. *Polq*^−/−^ mice have a mild phenotype: their erythrocytes had increased micronucleus frequencies but the gene knockout is not lethal [3]. However, combined defects in Pol θ and one of the HR factors (such as BRCA1, BRCA2, FANCD2, RAD51) lead to catastrophic consequences. Cells with impaired HR rely on TMEJ, and the disruption of both repair pathways results in cell death [31,57,58]. A comparable situation is observed in the cells with defects in another DSB repair pathway—c-NHEJ [28,47]. Moreover, recent CRISPR genetic screens identified 140 *POLQ* synthetic lethal gene partners, including genes related to DDR pathways distinct from DSB repair [57]. Mutations of *POLQ* in repair-deficient contexts increases levels of replication-associated DSBs, regardless of the initial source of DNA damage.

For example, in cells with a compromised HR pathway, the accumulation of cytotoxic RAD51-associated products is prevented by the anti-recombinase activity of Pol θ [19]. In BRCA2/RAD51-deficient cells, the DNA polymerase activity of Pol θ that fills accumulating gaps protects stalled forks from breakage under replicative stress [59]. Pol θ rescues the lethality associated with the impairment of Holliday-junction resolvases SLX4 and GEN1 through the suppression of error-prone mitotic crossing over in *D. melanogaster* [60]. In a similar vein, Pol θ can inhibit interhomolog recombination induced by DSBs [61]. With regards to c-NHEJ, the absence of the Ku protein significantly increases the engagement of Pol θ in DSB repair. Moreover, the inaccessibility of the 5′-ends and formation of collapsed forks preventing the loading of Ku proteins at DNA break ends can be resolved by TMEJ [28].

It was also shown that Pol θ exhibits DSB repair activity on collapsed forks induced by replication stress (e.g., under treatment by ATR and topoisomerase inhibitors resulting in SSBs accumulation) [62]. The involvement of Pol θ in replication restart of stalled replication forks following G-quadruplexes stabilization and UV-irradiation was also demonstrated [63,64]. In addition, TMEJ is a major mechanism of ICLs repair. In *Polq*^−/−^ MEF cells, mitotic crossovers were observed much more often under mitomycin C treatment than in the wild-type cells [57].

## 5. Beyond TMEJ: DNA Translesion Synthesis

The role of Pol θ is likely not restricted to DSB repair. Several biochemical and genetic studies suggest that Pol θ may be an important factor of TLS.

### 5.1. TLS Opposite Abasic Sites

Apurinic/apyrimidinic sites (abasic sites, AP sites) are the most common and potentially mutagenic DNA lesions arising as a result of spontaneous or enzymatic disruption of the glycosidic bond between the nucleobase and the sugar-phosphate backbone [65]. Pol θ bypasses AP sites with moderate efficiency by incorporating predominantly dAMP according to the “A-rule”. Unlike many other TLS polymerases, Pol θ demonstrates the subsequent polynucleotide strand extension, which resembles Pol ζ activity [51,66]. A large number of 1- and 2-nt deletions formed by the misalignment-misinsertion mechanism was also observed during TLS opposite an AP site [66]. Deletion mutants of Pol θ lacking the Insert 2 or Insert 3 lack the ability to bypass AP sites [16]. It was shown that the R2254 residue of the Insert 2 forms a salt bridge with the 3′-terminal phosphate of the primer and is required for the AP site bypass [11]. In addition, both tyrosine residues in 2387 and 2391 positions of the fingers subdomain are indispensable for AP site bypass in vitro [67]. However, the role of Pol θ in TLS opposite AP sites in vivo remains unclear.

### 5.2. TLS Opposite Oxidative DNA Lesions

Oxidative DNA lesions are formed by the attack of reactive oxygen species. The cytotoxic thymine glycol (Tg) and mutagenic 8-oxo-7,8-dihydroguanine (8-oxoG) are the most common oxidative DNA lesions [65,68]. The *POLQ*-knockdown cells have reduced viability after H_2_O_2_ treatment, suggesting that Pol θ might be involved in TLS opposite oxidative DNA lesions [69].

Pol θ efficiently bypasses both enantiomers (5R,5S) of Tg in vitro, preferentially incorporating complementary dAMP. However, primer extension downstream of the lesion is carried out in an error-prone manner [51,70]. Bypass product analysis also revealed 2-nt deletions, indicating the utilization of a misalignment-misinsertion mechanism. Similar to an AP site, Insert 2 (in particular, the R2254 residue) and Insert 3 are critical for the TLS activity opposite Tg [11,16].

A crucial role in the error-free bypass of Tg in human cells has been demonstrated to be attributed to the cooperative activity of Pol κ and Pol ζ. However, siRNA-mediated knockdown of these two DNA polymerases decreases the TLS by only 50%. It was shown that Pol θ is responsible for the relatively mutagenic bypass of Tg (compared to Pol κ/Pol ζ) in human fibroblasts [71]. Thus, Pol θ can be involved in an alternative Tg TLS pathway, taking on the role of both an inserter and an extender DNA polymerase.

A next-generation sequencing-based assay demonstrated that *POLQ*-deficient HEK293T cells possess decreased efficiency of 8-oxoG bypass [69]. However, the TLS activity of Pol θ opposite 8-oxoG has not yet been investigated in vitro. Among other oxidized lesions bypassed by Pol θ in vitro are AP-site derivatives: 2-deoxyribonolactone and C4′-oxidized AP sites [66].

### 5.3. TLS and Other Lesions

1,N^6^-ethenodeoxyadenosine (εA) lesion blocks the formation of the canonical Watson–Crick base pairing in DNA and severely inhibits most DNA polymerases [72]. Pol θ carries out relatively effective and error-prone TLS opposite εA in vitro, incorporating, predominantly, dAMP [73]. Analysis of siRNA-mediated knockdowns of DNA polymerases genes in mammalian cells identified a few independent pathways that promote replication through εA, including Pol θ-dependent mechanisms. Pol θ was shown to carry out more accurate replication across εA in cells compared to experiments in vitro [73].

It has been suggested that conserved tyrosine residues in the fingers are pivotal for efficient TLS opposite εA [67]. Substitutions of Tyr2387 and Tyr2391 with alanine significantly reduced the DNA polymerase activity on templates with εA in vitro. However, TLS assays in both human and mice cells revealed that the contribution of these tyrosine residues for εA bypass is ambiguous. Tyr2387 promotes mutagenic TLS, while Tyr2391 suppresses error-prone synthesis, and their epistatic interaction mitigates Pol θ mutagenicity. It has been proposed that protein–protein interactions and/or post-translational modifications can promote Pol θ active-site rearrangement, leading to the rotation of εA to a *syn*-conformation and binding complementary incoming dTMP via Hoogsteen interactions. In turn, Tyr2387 and Tyr2391 in purified enzymes support the extrahelical position of εA and Pol θ inserts non-complementary dAMP [67].

Pol θ plays a role in TLS opposite some alkylated DNA lesions. Pol θ along with Pol ν were demonstrated to be important for TLS opposite the O^6^-alkyl-2′-deoxyguanosine lesion in HEK293T cells. It was suggested that Pol ν operates as an inserter and Pol θ extends the primer downstream of the lesion [74]. Pol θ incorporates complementary dTMP opposite N^3^-methyladenine and is involved in one of the three independent TLS pathways opposite this lesion in human cells [75]. It was also demonstrated that Pol ι/Pol θ are responsible for a combined error-free bypass of N1-methyladenine in human cells [76].

Strikingly, Pol θ is crucial for inhibiting skin-cancer development via primary mutagenic TLS and other mechanisms of genome protection [63]. The frequency of skin tumors is higher in *Polq*^−/−^ mice. Experiments with siRNA-mediated knockdowns of different DNA polymerases in human fibroblasts suggested a few alternative pathways to bypass UV-induced lesions, where Pol θ is involved in mutagenic TLS. Pol θ-depleted human and murine cells have a significantly reduced survival, increased DSBs formation and chromosomal aberrations after UV treatment. Moreover, DNA fiber assays support the contribution of Pol θ to replication fork progression in UV-irradiated cells. However, biochemical studies showed the limited ability of Pol θ to bypass UV-induced lesions in vitro. Seki et al., demonstrated that Pol θ is unable to bypass both cyclobutane pyrimidine dimers (CPD) and 6–4 pyrimidine-pyrimidone photoproducts ((6–4) PP) but carries out the extension of the primer termini from a nucleotide opposite these lesions [77]. Another study demonstrated the ability of the PD of Pol θ to insert nucleotides opposite the 3′T of T^T CPD and to extend the primer from the 3′T of a (6–4) TT photoproduct [51].

## 6. Beyond TMEJ: Base Excision Repair

Many types of non-bulky DNA lesions including oxidative DNA lesions are fixed via base excision repair (BER). *POLQ*/*POLB* double-knockout DT40 cells demonstrate increased hypersensitivity to oxidative stress, suggesting that Pol θ may be a back-up protein in BER, where Pol β is the major player [78]. The molecular basis of this phenomenon is an intrinsic 5′-deoxyribose phosphate (dRP) lyase activity of Pol θ. Under certain conditions, Pol θ carries out single-nucleotide patch BER with low efficiency in vitro [79]. Unlike DNA polymerases of the X family, the active site of Pol θ possesses both the DNA polymerase and dRP lyase activities, and K2383 is crucial for both activities [79,80]. Considering the key role of DNA polymerase in TMEJ, D. J. Laverty et al., suggested that alternative substrates for the dRP lyase activity of Pol θ could be DNA lesions produced during DSB repair, such as AP sites on the 5′-overhangs [81]. The possible involvement of Pol θ in BER, as well as the ability of the active site to accommodate loops-out containing sequences, allows to propose a hypothesis about the role of Pol θ in Huntington’s disease (HD). This neurodegenerative disorder is characterized by trinucleotide repeat (TNR) expansion within the *HTT* gene under the oxidative stress. It was demonstrated that Pol θ efficiently extends CAG/CTG hairpin primers in BER-like substrates resulting in the expansion of TNRs in vitro. Moreover, cells derived from patients with HD contain significantly high levels of chromatin-bound Pol θ correlated with the degree of CAG repeat expansion [82].

## 7. Beyond TMEJ: RNA-Templated DNA Repair?

RNA can act as a template for DNA synthesis in the reverse transcription of retroviruses and retrotransposons, telomeric DNA synthesis as well as serve as a template for DNA synthesis during DSBs repair in yeast [83,84,85].

It was shown that Pol θ exhibits efficient reverse transcriptase activity, similar to classic retroviral reverse transcriptases (RTs) encoded by Moloney murine leukemia virus (M-MuLV) and avian myeloblastosis virus (AMV) [86]. Remarkably, Pol θ exhibits a significantly higher velocity and fidelity of DNA synthesis on RNA versus DNA. The RT activity of Pol θ is not unique among human DNA polymerases. Pol η and Pol κ from the Y family also possess RT activity [87,88] but it is less efficient relative to the RT activity of Pol θ [86]. Moreover, unlike Pol η, Pol θ strongly discriminates against incorporating ribonucleotides [86].

The crystal structure of Pol θ in a complex with the DNA/RNA primer-template and incoming deoxyribonucleotide demonstrated that (like retroviral RTs) Pol θ forms multiple hydrogen bonds with the 2′-hydroxyl ribose groups of RNA template [86]. Additional contacts of Pol θ with RNA may suppress template misalignment errors and contribute to its higher fidelity on RNA templates. Retroviral RTs do not exhibit significant structural transformation to accommodate the DNA/RNA hybrid. In contrast to RTs, the thumb subdomain of Pol θ undergoes a major structural transformation (57% residues refold from α helices to loops) to accommodate the thicker A-form DNA/RNA [86].

Ribonucleotides are the most common endogenous nucleotide base lesion in the eukaryotic genome [89,90]. The 2′-hydroxyl ribose group renders RNA ~100,000-fold more susceptible to spontaneous hydrolysis under physiological conditions [91]. Unrepaired ribonucleotides can cause DNA strand breaks, leading to genome instability [92,93]. It was suggested that Pol θ accommodates template ribonucleotides during DNA repair contributing to cellular tolerance of genome-embedded ribonucleotides. It was also demonstrated that Pol θ promotes RNA-templated DNA repair events detected by the GFP knock-in reporter assay in model mammalian cells. However, unlike yeast, the RNA-templated DNA repair has not yet been established in mammals. Thus, the biologically function of the RT activity of Pol θ is not clear and it requires future research.

## 8. How Attractive Is Pol θ as a Target in Cancer Therapy?

Unique biochemical properties and functions of Pol θ suggest that it is a prospective target for cancer treatment. To date, many studies have supported the association of Pol θ expression/activity changes with disease development and progression, increased resistance to chemotherapeutic agents and poor prognosis in patients with breast, liver, prostate, esophagus, colon, lungs, stomach and pancreas cancer [94,95,96,97,98,99,100,101,102].

Overexpression of *POLQ* is often detected in cancer cells. The increased expression is prominent in HR-deficient tumors, compensating for the impaired pathway. Nevertheless, the underlying mechanisms for changes in expression regulation are largely unestablished. One study reported suppression of *POLQ* expression by binding the transcription factor zinc finger E-box binding homeobox 1 (ZEB1) to the *POLQ* promoter in breast cancer cells [103]. Smolinska et al. described another mechanism of changing the enzyme expression. Oncogenic *KRAS* mutations, driving malignant transformation of pancreatic cells, markedly upregulate the expression of TMEJ factors Mre11, Lig3, and Pol θ. TMEJ activation in murine and human cells with *KRAS* mutations resulted in the promotion of pancreatic intraepithelial neoplasia. Since TMEJ is the most preferred DNA repair pathway in pancreatic ductal adenocarcinoma cells, inhibition of Pol θ led to a slowing of disease progression and improved survival of animal models [96].

**Figure 4:**
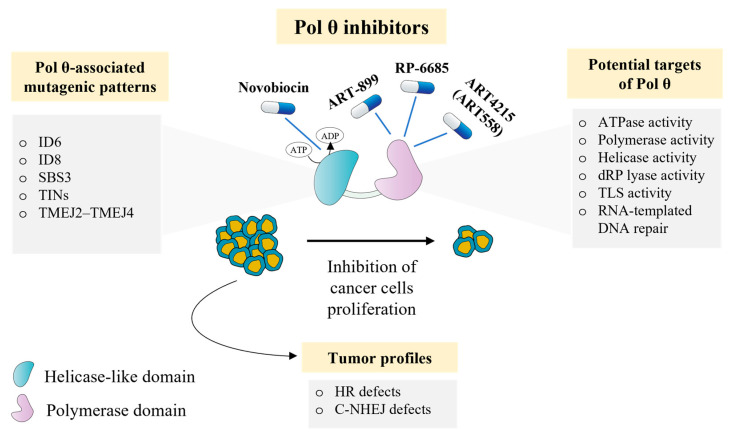
Pol θ as a target in cancer therapy.

Thus, Pol θ is not just a promising target, but already a real candidate for adjuvant therapy in pre-clinical and clinical studies (Figure 4). In some cases, off-target activities of drug compounds provide therapeutic benefits. A coumarin-derived antibiotic from *Streptomyces niveus* novobiocin is an example of drug repurposing. Novobiocin inhibits bacterial DNA synthesis by targeting DNA gyrase and DNA topoisomerase IV [104]. Novobiocin also selectively binds the HD of Pol θ, blocking the ATPase activity and inhibiting TMEJ. In HR-deficient cancer cells, this antibiotic exhibited an effective cytotoxic effect caused by the accumulation of ssDNA intermediates [105]. Among the on-target agents that selectively inhibit the DNA polymerase activity of Pol θ, orally bioavailable compound RP-6685 demonstrated its effectiveness on an HR-deficient mouse tumor xenograft model by inhibiting TMEJ and suppressing cancer-cell proliferation [106]. Another compound, ART558, identified by small-molecule screening, inhibits the DNA polymerase activity of Pol θ by binding the fingers subdomain [107]. Given the increased sensitivity of Pol θ-deficient cancer cells to ionizing radiation, combining Pol θ inhibitors with radiotherapy is also a promising therapy option. The application of ART558 and its more metabolically stable analogue, ART899, with radiation therapy demonstrated higher efficacy compared to radiation alone, as well as good tolerance in preclinical animal models [108]. In late 2022, ART558 (ART4215) had entered phase I/IIa clinical trials. The drug is intended to be administered both as monotherapy and in combination with PARP inhibitors (PARPi) in patients with HR-deficient advanced and metastatic solid tumors (ClinicalTrials.gov Identifier: NCT04991480) [58].

The pharmacological effect of Pol θ inhibitors is consistent with the findings reported in studies using synthetic lethality. Defects of the HR pathway are the main sensitivity factors to therapy based on Pol θ inhibitors [58,109]. Such drugs are expected to combine very effectively with PARPi, a class of drugs for BRCA1/2-deficient tumors. However, tolerance to PARPi is relatively widespread among cancer patients. Several studies have reported restoration of cancer-cell sensitivity to PARPi upon Pol θ deficiency [105]. Therefore, the combination of treatment with Pol θ and PARPi is quite promising but the mechanism of this phenomenon is not yet completely clear. Nevertheless, there are concerns about the possible long-term consequences of Pol θ inhibition due to its versatile nature, in particular, its ability to suppress genomic instability related to telomere length. In other words, telomere dysfunction associated with Pol θ insufficiency in cancer cells can increase tumor genetic heterogeneity and polyclonal drug resistance [110]. Moreover, ongoing studies have not investigated the effect of Pol θ-deficiency on other metabolic pathways, including TLS and BER [111].

It is also possible that levels of *POLQ* expression and unique mutation “footprints” (signatures) of Pol θ in tumor cells can be used as clinical predictors of the therapy efficacy or biomarkers of clinical outcome. Pol θ modulates resistance to docetaxel, cisplatin, and topoisomerase inhibitors forming DNA-protein crosslinks and ATR inhibitors [62,101,112,113]. The frequency of TINs formation has been shown to be higher in BRCA1/2-deficient tumors [34]. Analysis of mutations associated with increased *POLQ* expression in *BRCA*-mutated tumors resulted in the identification of new signatures: single-base substitution signature 3 (SBS3), small insertion and deletion signature 6 (ID6) and signature 8 (ID8) [114,115]. To improve the quality of diagnostic and clinical outcome prediction in patients receiving platinum-based chemotherapy for ovarian cancer, one of the studies refined novel mutational signatures based on the presence of deletions with different microhomology lengths (TMEJ2–TMEJ4), highly specific for tumors containing mutations in the *BRCA2* gene [116]. Moreover, approximately 30% of ductal carcinoma and lobular carcinoma breast cancers have genetic alterations in synthetic lethality *POLQ* gene partners and show enhanced Pol θ/TMEJ activity, thereby suggesting that Pol θ inhibition is a promising therapeutic strategy [57].

## 9. Conclusions

Pol θ is a unique DNA polymerase-helicase protein with a reduced requirement for a primer–template complementarity to carry out DNA synthesis. Intensive undergoing studies established the role of Pol θ in TMEJ during DSB repair and suggested the involvement of Pol θ in other DDR pathways. Although these pathways are potential mutagenic drivers, they protect the genome from serious alterations at the cost of possible minor changes. Recent studies improved the understanding of the structure of Pol θ and molecular mechanisms of TMEJ. However, many aspects of Pol θ functioning and regulation in cells are not clear yet (e.g., the mechanism of the nonhomology DNA ends trimming and the role of accessory regulatory proteins such as 9-1-1 and FANCD2 in TMEJ). The cryo-EM structure of the full-length Pol θ is also required for understanding of the TMEJ mechanism.

Recent progress in studies demonstrated that Pol θ is a promising target for the treatment of HR- or c-NHEJ-deficient cancers. Moreover, several off- and on-target inhibitors of Pol θ were identified. Nevertheless, there are some concerns about the application of Pol θ inhibitors: these are long-term effects associated with the development of tumor genetic heterogeneity, as well as the poorly understood influence of Pol θ-deficiency on TLS, BER, and other DDR pathways, given the multifaceted nature of the enzyme. Comprehensive future studies of Pol θ properties and functions will possibly overcome these limitations.

## Data Availability

Not applicable.
